# Improving the experience of facility-based delivery for vulnerable women through obstetric care navigation: a qualitative evaluation

**DOI:** 10.1186/s12884-021-03842-1

**Published:** 2021-06-11

**Authors:** Kirsten Austad, Michel Juarez, Hannah Shryer, Patricia L. Hibberd, Mari-Lynn Drainoni, Peter Rohloff, Anita Chary

**Affiliations:** 1 Wuqu’ Kawoq | Maya Health Alliance, 2da Avenida 3-48 Zona 3, Barrio Patacabaj, Tecpán, Chimaltenango, Guatemala; 2 Department of Family Medicine, Boston University School of Medicine, Boston Medical Center, 850 Harrison Avenue, Dowling 5, Boston, MA 02118 USA; 3 Department of Global Health, Boston University School of Public Health, 715 Albany Street, Boston, MA 02118 USA; 4 Section of Infectious Disease, Department of Medicine, Boston University School of Medicine, 801 Massachusetts Avenue, Boston, MA 02118 USA; 5 Department of Health Law Policy & Management, Boston University School of Public Health, Boston, USA; 6 Evans Center for Implementation and Improvement Sciences, Department of Medicine, Boston University School of Medicine, Boston, USA; 7 Division of Global Health Equity, Brigham and Women’s Hospital, 75 Francis Street, Boston, MA 02115 USA; 8 Department of Emergency Medicine, Brigham and Women’s Hospital, 75 Francis Street, Boston, MA 02115 USA

## Abstract

**Background:**

Global disparities in maternal mortality could be reduced by universal facility delivery. Yet, deficiencies in the quality of care prevent some mothers from seeking facility-based obstetric care. Obstetric care navigators (OCNs) are a new form of lay health workers that combine elements of continuous labor support and care navigation to promote obstetric referrals. Here we report qualitative results from the pilot OCN project implemented in Indigenous villages in the Guatemalan central highlands.

**Methods:**

We conducted semi-structured interviews with 17 mothers who received OCN accompaniment and 13 staff—namely physicians, nurses, and social workers—of the main public hospital in the pilot’s catchment area (Chimaltenango). Interviews queried OCN’s impact on patient and hospital staff experience and understanding of intended OCN roles. Audiorecorded interviews were transcribed, coded, and underwent content analysis.

**Results:**

Maternal fear of surgical intervention, disrespectful and abusive treatment, and linguistic barriers were principal deterrents of care seeking. Physicians and nurses reported cultural barriers, opposition from family, and inadequate hospital resources as challenges to providing care to Indigenous mothers. Patient and hospital staff identified four valuable services offered by OCNs: emotional support, patient advocacy, facilitation of patient-provider communication, and care coordination. While patients and most physicians felt that OCNs had an overwhelmingly positive impact, nurses felt their effort would be better directed toward traditional nursing tasks.

**Conclusions:**

Many barriers to maternity care exist for Indigenous mothers in Guatemala. OCNs can improve mothers’ experiences in public hospitals and reduce limitations faced by providers. However, broader buy-in from hospital staff—especially nurses—appears critical to program success. Future research should focus on measuring the impact of obstetric care navigation on key clinical outcomes (cesarean delivery) and mothers’ future care seeking behavior.

**Supplementary Information:**

The online version contains supplementary material available at 10.1186/s12884-021-03842-1.

## Background

Recently, quality of care and patient experience have become key targets of global efforts to reduce maternal mortality [1–3]. Skilled birth attendance—the primary strategy to prevent maternal deaths in low-resource settings [4]—requires that mothers be willing to seek care, can get to maternity facilities in a timely manner, and receive quality care upon arrival. A growing body of research describes the profound negative impact that disrespectful and abusive care has on each of these steps in the referral chain [1, 5–7]. Negative patient-provider interactions—including physical abuse, non-dignified care, and non-consented care—lead mothers to fear, delay, or refuse in-hospital delivery. Importantly, drivers of disrespectful and abusive care extend beyond the interpersonal level. At the facility level, material deficiencies and overcrowding in hospitals prevent providers from delivering quality care. At the societal level, larger forces—including discrimination on the basis of ethnicity and economic status—threaten mothers’ confidence in public hospitals [8]. Understanding the vital link between quality of care and maternal mortality, the World Health Organization (WHO) has put forth universal standards of maternity care that address key domains across these three levels [3].

Indigenous mothers suffer a disproportionate burden of maternal mortality worldwide and are one of the groups most vulnerable to disrespectful and abusive care [9–12]. For example, in Guatemala, a Central American country where approximately half of the population is of indigenous descent, while government-sponsored health care, including prenatal care and facility-based delivery, is free of charge to Guatemalan citizens, stark health disparities exist. Indigenous Maya mothers are nearly twice as likely to die from childbirth as their non-indigenous counterparts in Guatemala [13]. Only 15.8% of indigenous Maya mother give birth in a facility; the majority instead deliver at home with traditional midwives, who typically learn through a combination of apprenticeship and formal training through the Ministry of Health and integration both traditional practices—such as prenatal massage and postpartum temezcal (sweat lodge)—with biomedical prenatal care practices [14–17]. 

The prevalence of home delivery is due in part to cultural norms and preferences. Women may enjoy the social support offered by family members during home delivery, while in some cases their agency to elect hospital delivery may be constrained by the opinions of male partners and their families [18–20]. Yet, disparities in maternal mortality are also shaped by economic, geographic, and linguistic barriers to care, as Maya mothers living in rural areas have limited access to health care facilities, where care is provided in Spanish rather than in indigenous languages, and may face disrespectful and abusive care upon arrival [18, 21]. One strategy to improve rates of skilled birth attendance among Maya mothers in Guatemala is to create alternative sites with some features of home delivery where basic emergency obstetric care can be provided, known as Casas Maternas (maternal waiting houses) [22]. In other low-resource settings, hospital-level interventions, such as staff training, have been deployed to reduce disrespectful care [23].

We have recently published about an alternative approach—obstetric care navigation—to decreasing referral barriers by providing accompaniment to Maya mothers planning to deliver at home with traditional midwives who experience obstetric complications [24]. Obstetric care navigators (OCNs) are a hybrid community health worker who collaborate with traditional midwives to facilitate referrals. OCNs function similarly to care navigators who help mothers overcome referral barriers, an approach shown to improve cancer care outcomes in both low- and high-income settings [25–27]. OCNs also offer one-on-one labor support (similar to doulas), inspired by evidence from high-resource settings that it can lead to shorter labor, lower rates of cesarean delivery, and improve patient experience [28].

We rolled out of the first ever OCN pilot project beginning in April 2017 and recently published initial quantitative results showing marked improvements in facility-level birth rates [29]. In the current study, we sought to better understand patients’ and providers’ experiences of obstetric care navigation through qualitative interviews with mothers who received care navigator accompaniment and hospital staff at referral centers, specifically physicians, nurses, and a social worker. The goals of this study were to [1] identify barriers to facility delivery among Maya mothers and to the hospital staff caring for them [2]; characterize the OCN roles mothers and hospital staff valued most and how these mapped onto the WHO Standards of Maternal and Newborn Care [3]; and [3] identify the impact of OCNs on mothers’ facility birth experience (including potentially negative impacts).

## Methods

### Description of the intervention

The theory, design, and outcomes of the OCN pilot launched in April 2017 have been published in detail elsewhere [24, 29]. Briefly, the pilot evaluated by this qualitative research was implemented by Maya Health Alliance, a non-governmental organization providing primary health care to rural Maya villages. OCNs were local Maya women bilingual in Spanish and Maya Kaqchikel recruited from local villages into a salaried position for the duration of the project. Their charge was to bridge the weak referral chain for mothers with obstetric complications between homebirths and hospital deliveries by coordinating logistics, reducing patients’ reluctance to referral, and facilitating medical care in public facilities. OCNs were trained in labor support, language interpretation, and conflict de-escalation. They met with project staff in bi-monthly quality improvement meetings to review patient cases, debrief difficult encounters, review project progress, and make small iterative improvements to the program.

A total of 41 traditional midwives were supported by Maya Health Alliance participated (all of whom were formally registered with the Ministry of Health), and all patients under their care were eligible for OCN accompaniment. Patient referrals were generated from routine midwifery assessment and with the help of an interactive smartphone application [30]. When need for emergency referral was detected, traditional midwives contacted the Maya Health Alliance 24/7 emergency line and the on-call OCN was assigned to the case. The OCN first helped by coordinating referral logistics, including emergency transportation via public ambulance services (when available) or private vehicle (with project funding). Four facilities (two primary, one secondary, and one tertiary care center) run by the Guatemalan Ministry of Health were within the catchment area of the affiliated traditional midwives. Medical directors and leadership of the obstetrics departments were engaged to provide input on OCN design, to arrange logistics (such as acceptance of OCNs in clinical areas), and to establish lines of communication regarding references and barriers encountered.

### Study design and sampling

For the current analysis, we conducted interviews with patients who received OCN services. Patients were recruited from a list generated by project staff of mothers age 18 or older who received OCN accompaniment for a significant obstetric event (emergency referral, hospital birth, or adverse birth outcome) in the first 9 months of the program (April–December 2017) to include a diverse mix of birth experiences (ex: vaginal and cesarean delivery) and outcomes (ex: neonatal complications). The list of approximately 40 was randomized and mothers were visited sequentially at home by study staff until the enrollment goal of 17 patients was reached; all agreed to participate. Enrollment goal was based on prior research suggesting saturation at 12 interviews [31].

We also interviewed practitioners in collaborating MoH facilities who were recruited through purposeful sampling. The directors of obstetrics provided a list of approximately 35 resident and attending physicians and nurses likely to have had contact with OCNs during the first 8 months of implementation. Each was approached in a random order and offered the chance to participate until the goal of four participants in each group was reached, with plan to pursue further interviews if saturation was not reached within each sub-group. All those invited to participate agreed. The sole social worker for the hospital was also interviewed given her frequent interaction with OCNs during routine patient care. In total, 13 hospital staff members were interviewed.

### Data collection

Two Guatemalan female anthropologists conducted semi-structured interviews between January and March 2018. Interviews were conducted in a private space in the subject’s preferred language (Spanish or Maya Kaqchikel) and lasted 25–50 min. Each interview was recorded and transcribed verbatim. Those in Kaqchikel were then translated into Spanish by a linguist. Transcripts were redacted to eliminate identifying information. Limited demographic data was also collected from patients (including parity, number of living children, location of most recent birth and mode of delivery) and hospital staff (including current professional title and number of years at current hospital).

Interviews with both patient and hospital staff focused on several topics (full guide in Additional file 1). First, we examined perceived motivators of home birth with traditional midwives and barriers faced by Maya mothers to facility-level obstetric care. Next, questions probed interviewee’s understanding of OCN’s intended purpose and activities, the intervention’s impact (both positive and negative) on clinical care, and the experience of patients and providers. Problems left unaddressed by the OCN intervention were also solicited. Additionally, patients were asked about their personal interaction with OCNs and whether their accompanied referrals changed their perception of the public health system. In addition to the above, health workers were asked about what systems-level barriers exist to providing quality care to Maya mothers and any perceived impact of OCNs on hospital culture.

### Data analysis

We analyzed interview transcripts and notes using an inductive approach and a constructivist paradigm, in which researchers privilege and seek to understand participants’ views of the situation being studied [32]. Two authors (KA and HS) developed a preliminary codebook upon review of the first two transcripts in each category, which was then reviewed and revised by the senior author (AC) in three rounds. Two of three bilingual authors (KA, HS, and AC) coded each transcript independently in Spanish. Discrepancies were resolved by the third coder. Team members serially reviewed the coded data until consensus on the major themes was reached. Discrete OCN activities were abstracted and organized into domains selected through author discussion. Lastly, interviewee statements that exemplified one of the seven non-clinical domains of the WHO Standards of Care were abstracted. Coding was facilitated by NVivo 11 software (QSR International; Melbourne, Australia). This study was approved by the Institutional Review Board of Maya Health Alliance (protocol WK-2017-008). Results are reported using the Consolidated criteria for reporting quality research checklist [33].

Quantitative data was summarized as medians given lack of normal distribution.

### Reflexivity

Qualitative researchers recognize that their personal values and experiences can influence the research process and data interpretation, referred to as reflexivity. Our study team members included one Guatemalan physician (MJ), three U.S.-trained physicians with over a decade of clinical and research experience in rural Guatemala (KA, PR, AC), a U.S.-trained researcher (HS), a U.S.-trained expert in global maternity care (PH), and two U.S.-trained researchers with expertise in qualitative methods (AC, MD). Interpretation of results herein is based on group conversations.

## Results

Characteristics of interviewees are presented in Table 1. Among patients interviewed most (15/17) gave birth at a hospital with OCN accompaniment, with ten cesarean and five vaginal deliveries. Two mothers (12.5%) delivered vaginally at home with traditional midwives after receiving hospital referral for complications earlier in pregnancy. Their median parity was 3.2, excluding the seven mothers without a prior delivery (41.2% sample). All four resident physicians interviewed were completing years one and three out of 4 years of training. Nurses worked at the tertiary care hospital for a median of 13.3 years; for attending physicians, the median was 8.8 years.

**Table 1:** Demographic information of patients and hospital staff participants in qualitative interviews

Characteristics	
***Patients***	
Nulliparous (#)	7
Multiparous (#)	10
• Parity (median)	3.2
• Living children (median)	2.5
Language of interview	
Spanish (#)	9
Kaqchikel Maya (#)	8
Place of delivery	
Hospital (#)	15
• Cesarean (#)	10
• Vaginal (#)	5
Home (#)	2
***Hospital staff***	
Resident physicians (#)	4
Senior (attending) physicians (#)	4
Nurses (#)	4
Social worker (#)	1

Comments from interviewees fell into three categories: existing barriers to hospital delivery, perceived benefits of OCNs, and critiques of OCNs. Each of these themes is described below.

### Existing barriers to hospital delivery

Mothers cited fear and isolation as the most common reasons to avoid or refuse referrals to public obstetric facilities. Patients contrasted hospital delivery to home birth, in which they have emotional support of family and traditional midwives. Patients’ specific fears included inability to communicate fully with providers, abuse (most commonly verbal), and unconsented care. Fears stemmed from both personal experiences during prior pregnancies and community opinion and stories. One mother stated:

*“They say that the hospital is not a good place, that they kill people there.” (Patient 33)*

Nine of 17 interviewees offered that they were fearful of being “operated on” at public hospitals, a phrase referring to cesarean delivery, surgical sterilization, or both. Among mothers who stated a preferred mode of delivery, all (12/12) reported wanting a vaginal birth. Reasons for this preference included general fear of surgery, desire to delivery at home where family could care for her postpartum, and quicker recovery time of vaginal delivery which allowed for quicker return to domestic responsibilities.

Providers identified two cultural factors that they perceived led to mothers’ resistance to facility delivery. The first of these was male-dominated (“*machista*”) culture, in which the mother’s health was not a priority of male partners. The second regarded patients’ spiritual beliefs. For example, one resident physician reported:

*“The Maya have their cosmovision. As such, they see everything as related to Mother Nature, their grandparents, their ancestors, and this way of thinking is what makes them not want to come to the hospital.” (HCW19)*

Patients did not identify any spiritual beliefs as a barrier to hospital-level care.

Providers recognized that rumors of mistreatment and fear of overzealous surgical intervention led mothers to avoid hospital referral, but felt they were overexaggerated. Physicians cited overcrowding, inadequate resources, and understaffing as drivers of these negative community perceptions. One attending physician described the link between these factors and mistreatment:

*“You have to understand that we are doctors, but we are also human beings. If I have been on call, did not sleep, did not eat dinner or breakfast, how can I be human? . . . Sometimes mistreating a patient, scolding, or not explaining something well is because of this.” (HCW 24).*

This frames health system deficiencies as negatively impacting both providers, through overwork, and patients, through unempathetic care resulting from physicians’ physical and emotional exhaustion.

### Perceived benefits of obstetric care navigation

Overall, mothers had positive experience with OCNs and found they reduced fear of hospital care. Similarly, physicians felt that OCNs improved both patient and provider experience. Opinions among nurses were mixed. Patients and providers identified four key beneficial OCN roles: communicate, coordinate, support, and advocate. Table 2 summarizes the specific activities associated with each role.

**Table 2:** Example activities obstetric care navigator identified by mothers, physicians, nurses, and social workers

	Obstetric Care Navigator Tasks
**Coordinate**	Navigate complex hospital workflows
	Facilitate transfer between levels of care (primary to tertiary referral)
	Bring prior labs and imaging results
	Purchase medications and supplies stocked out of public system
	Bring patient samples to outside laboratory (if not available in public system)
	Coordinate transportation from rural villages to hospital
**Communicate**	Language interpretation between mothers and medical providers
	Cultural interpretation between mothers and medical providers
	Contextualize limitations faced by medical providers for mothers and families
	Provide updates to family and traditional birth attendant
**Support**	Continuous labor support (e.g. verbal encouragement)
	Coach patient through uncomfortable procedures (e.g. cervical exam)
	Companionship during hospitalization
	Emotional support (e.g. express empathy, reassurance)
	Take steps to protect patient dignity (e.g. shielding mother while she changes into a gown in public labor ward)
**Advocate**	Alert nurses and doctors to mother’s needs
	Encourage mothers to accept necessary interventions (ex: cervical exam)
	Speak on behalf of mothers (e.g. reinforce refusal of Cesarean delivery)
	Prevent or deflect mistreatment

Specific obstetric care navigator roles identified through qualitative interviews (right column) fell into four broad categories (left column)

#### OCN role: communicate

Kaqchikel Maya-speaking patients valued OCNs’ ability to interpret between Kaqchikel and Spanish with Spanish-speaking providers, which allowed them to articulate questions and reduce fear of medical encounters. Mothers felt that OCNs prevented verbal abuse by making it easier for providers to talk with patients:

*“I was very afraid to go back [to the hospital] because there are doctors that scold [you], but I don’t understand what they are saying. Perhaps that is why they scold [you]. But not when I was accompanied by [the OCN]—she had the words to explain to them.” (Patient 1).*

This patient drew a direct connection between linguistic barriers and disrespectful and abusive maternity care, a theme repeated by other interviewees (see Table 3).

**Table 3:** World Health Organization Standards of Obstetric and Newborn Care and corresponding obstetric care navigator roles

	Description of WHO Standard	Example quotation about OCN	OCN Role
Standard 2	The health information system enables use of data to ensure early, appropriate action to improve the care of every woman and newborn	“*The OCNs tell me, ‘Doctor, she is pregnant based on this last menstrual period, she has not received an ultrasound, she already saw the nutritionist’*” (HCW 14)	Coordinate
Standard 3	Every woman and newborn with condition(s) that cannot be dealt with effectively with the available resources is appropriately referred	“*The [OCN] accompanies the women referred from rural villages, they support them to get to the hospital which to them is an unknown place*.” (HCW 23)	Coordinate
Standard 4	Communication with women and their families is effective and responds to their needs and preferences	*“It is hard, when I hear what the hospital staff are saying I don’t understand. In comparison, the OCN can explain well exactly what it is that they want me to understand.*” (Patient 11)	Communicate
Standard 5	Women and newborns received care with respect and preservation of their dignity	“*Last delivery there was a doctor who scolded me for having too many children*. *.. Because the OCN was with me they do not dare to scold me, they have to provide better care*.” (Patient 1)	Communicate Advocate
Standard 6	Every woman and her family are provided with emotional support that is sensitive to their needs and strengthens the woman’s capability	*“[The OCN] is such a help. It is like having a doctor, a midwife, and a friend close by. It’s like a home birth with my mother there. I think she is a big help.”* (Patient 4)	Support
Standard 7	For every woman and newborn, competent, motivated staff are consistently available to provide routine care and manage complications	“*The OCNs are very attentive to their patients’ needs. They will help them walk to the bathroom, because many times nursing has too few staff to help patients.*” (HCW 22)	Support Advocate
Standard 8	The health facility has an appropriate physical environment, with adequate water, sanitation and energy supplies, medicine, supplies and equipment for routine maternal and newborn care and management of complications	“*The OCNs have done a lot to facilitate patient care. They will bring samples to the outside laboratory and whatever we need we can depend on them*.” (HCW 13)	Coordinate

Mothers who reported speaking to their OCN in Spanish (*n* = 9) still found value in the OCN as an intermediary. The OCN’s role extended beyond linguistic interpretation, as also noted by one resident doctor:

*“It is often hard to convince the patients to stay, and even when we explained it clearly. Often, I can’t do it well. In comparison, the OCN can communicate better with the patient. She can explain things in a better way and help them understand what is happening.” (HCW 13*).

Here, the OCN translates medical information into lay terms and also acts as a trusted third party. Both patients and providers appreciated the OCN’s role in updating families.

#### OCN role: coordinate

Patients valued OCNs help in navigating the hospital environment unfamiliar to most mothers. They felt that accompaniment not only reduced fear and uncertainty but also facilitated timely care from hospital staff. One woman who had never been to a hospital before described:

“*When I arrived at the hospital, I didn’t even know where to begin, where to go and when. But when [the OCN] arrived, she explained, ‘first we get a medical chart, then we wait in this line to see the doctor.’ She helped so much because I did not know what to do” (Patient 7).*

Overall, doctors saw care coordination as the greatest advantage of OCNs. They noted multiple weaknesses in the referral process improved by OCNs’ presence. For example, patients are expected to retain all of their own test results as a personal medical record. OCNs provided continuity by transmitting information between providers at different levels of the public health system and carried medical records, which patients otherwise often forgot. A resident physician reflected:

*“The [OCN] can tell me the patient’s last period, if an ultrasound was done, if she saw the nutritionist. And I can say to her, ‘Please make sure the patient gets the biophysical profile and transvaginal ultrasound done,’ outside the hospital when we don’t have it here’” (HCW 14).*

As a result, this physician felt she could make better informed medical decisions.

#### OCN role: support

Overall, patients thought the most valuable function of OCNs was the emotional support during hospitalization. Patients described feeling “protected” or “safe.” A multiparous participant compared her prior pregnancy experience to her current OCN accompanied referral:

*“In the hospital no one helps you, as compared to a normal home birth where there is a lot of support . . . The nurse left me in the bed and said, ‘if you want to get up, do it yourself.’ What is needed in the hospital is help. The OCN helped me get out of bed” (Patient 31).*

The positive role filled by the OCN highlighted what some patients perceived as shortcomings in nursing care.

Patients also highly valued doula-like support:

*“[The OCN] told me to stay calm, that what I was experiencing [during labor] was normal. She told me not to be afraid, and that she was there by my side. If I needed medical help, she would go get the doctor” (Patient 29).*

Providers also saw the value in OCNs’ labor support and felt it allowed them to dedicate time to other urgent issues while still allowing patients to feel emotionally supported. One physician (HW 14) noted that OCNs often helped compensate for nursing shortages that negatively impact patient experience.

All patients who were asked (15/15) said that they would agree to a hospital referral in a future pregnancy. However, for more than half (9/15) acceptance was contingent on the OCN:

*“I don’t want to go alone. When [the OCN] accompanies me, I feel protected” (Patient R4).*

One physician felt that the OCN presence would have a positive impact on community perceptions of public maternity facilities:

*“I think that the women feel supported and will transmit that information to other women in their communities. They will say ‘at least there is someone there with mothers, someone who is dedicated to her’” (HW R14)*.

Other health workers expressed similar sentiments that positive experiences in hospital care enabled by OCNs could gradually change negative community opinions about these facilities.

#### OCN role: advocate

Patients reported OCNs performed both passive and active forms of advocacy. Patients felt that the presence of OCNs was an effective deterrent to mistreatment. For example, when asked if she noticed a different between accompanied and unaccompanied mothers one patients stated:

*“Yes, there is a difference. The other ones they scold.” (Patient 2)*

Other mothers were similarly thankful to have someone who spoke “on [their] behalf.” The phrase referred both to translation between languages, for Kaqchikel-speaking mothers, and phrasing medical facts in a form intelligible to those with low medical literacy. This sentiment extended to OCNs supporting patients’ right to refuse undesired surgical intervention. One mother who was referred to the hospital for decreased fetal movement and ultimately went on to an uncomplicated vaginal delivery recounted her experience of refusing Cesarean delivery:

*“I was very scared because they told me they would operate on me, but I did not want that. They were about to operate when the [OCN] helped me stop them. Thank God for her.” (Patient 35)*

In contrast, physicians saw OCNs as a means to facilitate acceptance and expediency of clinical interventions:

*“The patients usually don’t cooperate with us in the moment we need to examine them or do a procedure. Sometimes, we get behind seeing patients because we need someone to facilitate the exam. The OCNs can do that.” (HCW 20)*

More interviewee statements are presented in Table 3 organized according to the seven non-clinical WHO Standards of Care.

The World Health Organization’s Standards of Obstetric and Newborn Care (columns 1 and 2) are matched OCN activities identified through qualitative interviews with mothers, physician, and nurses (column 3). Column 4 links the Standards to the four categories of OCN roles identified through qualitative analysis. Of note, Standard 1—relating to use of evidence-based practices—is not included in the Table as this could not be evaluated through qualitative methods.

### Critiques of obstetric care navigation

Some health care workers viewed OCNs as able to perform clinical roles, and expressed disappointment when they did not. Two physicians found value in OCNs performing clinical tasks, and requested that OCNs act as assistants in the operating room, for example by handing the surgeon instruments. The tone of nurses’ comments were more critical. For example, some nurses expressed the view that OCNs would be more valuable if they instead performed nursing tasks:

*“If she [the OCN] is caring for her patient, she should help bathe her. But they are not authorized to. I see them just standing there, not helping” (HCW 18)*.

*“We cannot tell [OCNs] ‘change this patient’ or ‘look at her wound’ or ask ‘what are her vital signs?’ I think they could be trained to recognize warning signs, but I think that is better left to [nurses]” (HCW 17).*

The latter quote exposes a possible tension between the nurse’s desire for OCNs to help alleviate her patient care burden and being replaced by a new health worker.

The only drawback to the OCN model identified by patients was that they were not always available. For example, OCNs did not follow patients postpartum, though mothers reported unempathetic treatment by nurses during that time. Another interviewee recounted a referral during which the OCN who had previously accompanied her was not available (due to a training activity):

“*When the [OCN] is not there it isn’t the same quality of service. No one speaks for me, and I felt that no one helped me like I deserve to be helped. It is so difficult for me in the hospital without her.” (Patient 9)*

## Discussion

Obstetric care navigation is an innovative model to facilitate obstetric referrals and improve patients experience with facility-level obstetric care. Here we present qualitative interviews with mothers and hospital staff involved in the pilot OCN intervention, which increased facility delivery by more than two-fold [29]. Several themes emerged from interviews that inform the mechanism behind this improvement. First, mothers most valued the emotional and labor support roles given nearly universal patient fear and anxiety from the hospital environment. Second, mothers recognized the communication and advocacy roles of OCNs as deterrents of disrespectful and abusive care and undesired interventions. Third, physicians most valued that care coordination aspect of OCNs, in addition to reducing patient-provider communication barriers.

Our findings consistent with a study by Colombara and colleagues who examined determinants of satisfaction with skilled delivery among a large number (*n* = 10,895) of Indigenous mothers from Guatemala, Mexico, and Panama and found that the strongest predictors of satisfaction with maternity care were provision of care in Indigenous languages and being allowed accompaniment (family or traditional midwife) at hospital delivery [12]. Both of these were integral functions of OCNs identified by our participants. Many participants valued the OCNs’ ability to “speak on behalf” of them. Their descriptions imply more than language interpretation, but a broader ability to articulate their needs and preferences, overcoming the many competing priorities in the hospital environment and power differentials between poor Indigenous mothers and physicians and nurses.

Homebirths remain the norm in many LMICs, including Guatemala’s Maya communities [18, 34–36]. Mothers we interviewed articulated a need for personalized support during labor and delivery (such as help walking to the bathroom) that are missing from the typical hospital environment in low-resource settings. They drew direct parallels between the emotional and personal care provided by OCNs and that offered by family and traditional midwives during homebirths (see Patient 4, Table 3). These findings support the role of OCNs in meeting the WHO Standards of Care 4 through 6 (Table 3). If universal facility-based delivery is to be achieved, maternity hospitals must find a way to attend to the human and emotional needs of mothers, or patients will likely continue to choose homebirth despite the risks.

Our study participants also recognized OCNs’ role in mitigating disrespectful and abusive care, a problem recognized in maternity care worldwide [5, 6, 11, 37–39]. Mothers and hospital staff in our sample noted that OCNs effectively addressed abuse through multiple mechanisms, including deterrence (observer effect) and prevention of triggers for abuse, such as provider frustration from communication barriers. A benefit of the OCN model identified by these qualitative interviews is the direct targeting of physical and emotional overburdening of public hospital staff, which is a significant but underappreciated contributor to disrespectful and abusive care [40]. In contrast, strategies to reduce mistreatment of mothers to date have largely focused on education of hospital staff. For example, a project in Kenya combined training providers in respectful maternity care and improving governance and accountability in health facilities, and found this approach was associated with a reduction in most types of disrespectful and abusive care [23]. To achieve the greatest impact, future research should test the impact of combining OCN support with these strategies to create a multi-level intervention.

Most of our postpartum mothers in the study reported that fear of cesarean delivery was a deterrent to facility-based delivery and none voiced a preference for cesarean delivery. Surveys conducted in both high- and low-resource countries that have shown mothers overwhelmingly prefer vaginal delivery [41, 42]. Nonetheless, the rate of cesarean delivery has tripled over the past three decades globally [43]. A recent study from Guatemala—which included the catchment area for the OCN pilot described here—found that that cesarean delivery increased by 50% in the 3 year span from 2015 to 2017 [44]. Labor support is one of the very few interventions shown to decrease cesarean delivery rates, yet it is not routinely available in LMICs [45, 46]. Our OCN pilot demonstrated the feasibility of providing labor support in a low-resource public hospital, but the quantitative evaluation was not designed to detect changes in cesarean delivery rates pre- and post-implementation [46, 47]. In the qualitative interviewers analyzed here, mothers provided two mechanisms by which OCNs may decrease cesarean delivery: first, by offering intrapartum support, such as techniques to cope with pain and encouraging position change, that promote vaginal delivery, and second, by emphasizing mothers’ preferences for vaginal delivery to obstetricians. An important caveat, however, is that some providers in our study viewed OCNs as a means to convince patients to accept interventions. Interpersonal dynamics must be closely monitored to ensure OCNs are not tasked with compromising mothers’ reproductive freedoms.

While few criticisms emerged, qualitative interviews did identify important areas for programmatic improvement. Physician buy-in to the program was high, but nurses voiced desires for extra clinical help to lessen their workload. Multiple studies in LMICs have identified how unrealistic workloads and limited hospital resources can contribute to hospital staff’s mistreatment of and poor attitudes towards patients [37]. Similarly, we believe negative opinions expressed by nurses may be the result of exhaustion from clinical and emotional work, especially as the failures patients identified were in the emotional caring domain. After our initial review of these qualitative interviews, we made further efforts to identify nurse champions of the OCN program within the referral hospital. Increasing nursing engagement will be an important component of future implementation efforts.

### Limitations

One limitation of this study is that we did not conduct interviews with health care workers in the public community health centers that refer patients to hospitals. As such, their perspectives on the OCN program are not represented. In addition, patients may have underreported criticisms of the program for fear of not receiving OCN support in future pregnancies. Third, our recruitment of hospital staff favored those who had frequent interaction with OCNs and occurred during the initial phase of the pilot project. Opinions are likely to evolve over time, and longitudinal impressions of OCNs are not reflected in this manuscript. Fourth, women's agency in making decisions regarding reproductive health is often constrained b the patriarchal social structure common in rural Guatemalan communities, however for this study we chose to focus on maternal perceptions of OCNs instead of including family members in interviews [19, 20, 48]. Finally, an important difference between our obstetric care navigation program and other labor support models is that ours does not focus on patient education, which may limit direct comparison [28].

## Conclusions

Obstetric care navigation holds the potential to tackle two of the biggest goals in global obstetrics: increasing skilled birth attendance and optimizing the use of cesarean delivery. Through qualitative interviews we found that both patients and hospital staff valued the addition of OCNs to the obstetric care team for Indigenous mothers planning to deliver at home with traditional midwives. The model offers the unique benefit of addressing supply and demand-side barriers to quality care in low-resource public hospitals. As a pilot focusing on villages in rural Guatemala, structured implementation evaluations will be crucial to understand how OCNs function across diverse contexts and to identify the core programmatic elements around which local adaptation should take place [49, 50]. As the program continues today in a more limited scope, an encouraging note is that its success has not been threatened by multiple leadership changes in the affiliated public hospitals nor by the significant barriers posed by the COVID-19 pandemic. Future research on the OCN model should work to measure OCN’s impact on cesarean delivery rates, maternal and neonatal health outcomes, and maternal satisfaction with care, since empowering mothers is at the core of the OCN mission.

## Supplementary Information

**Additional file 1.** Patient and hospital staff interview guides (English version).

## Data Availability

The dataset used during the current study are available from the corresponding author on reasonable request.

## Abbreviations

LMIC: Low- and middle-income countries
OCN: Obstetric care navigator
WHO: World Health Organization
