# Supplementary material for: Improving the experience of facility-based delivery for vulnerable women through obstetric care navigation: a qualitative evaluation
Source: BMC Pregnancy Childbirth. 2021 Jun 11;21:425. doi: 10.1186/s12884-021-03842-1 (PMC8193958; doi:10.1186/s12884-021-03842-1)
Supplement: Supplementary file 1 — Additional file 1. Patient and hospital staff interview guides (English version). [file 12884_2021_3842_MOESM1_ESM.docx]

**Patient Interview Guide**

Obstetric Care Navigator Pilot Program Qualitative Study

Interview date:

Interviewer:

Language:

Consent to interview obtained?

Recorded or written?

I appreciate your willingness to talk with me today about your pregnancy experience. Remember that I will not write down your name with your answers so everything you say will be confidential. In general, we want you to provide specific examples whenever possible but not the names of specific people involved in that example.

1. Can you tell me a little about yourself?

- How old are you?
- What village do you live in?
- What is your village like?
- Were you born in this village? If not, where is your family from?
- Do you have any hobbies or activities you do to make money, such as weaving?

*Start recording here if patient agreed*

1. Can you tell me a little about your family?

- How many children do you have?
- Who helps you care for your children?

1. How many times in total have you been pregnant?

- *If recent birth is only pregnancy, skip to next question*
- *If more than one pregnancy, continue with probes below:*
  - Did you see someone for prenatal care during your previous pregnancies?
    - If so, who?
    - Did you feel good about the care you received? Why or why not?
  - Did you go to the hospital (CUM or national hospital) during these pregnancies?
    - Why did you need to go?
    - If so, where?
  - What was your experience like in CUM/National hospital?
    - Were you able to communicate with the doctor or nurse?
    - Did you understand the care or recommendations your received?
      - If not, did you feel able to ask questions?
    - Did you feel any negative feelings, such as fear or being judged?
    - Did you feel any positive feelings, such as being supported?

1. Have you heard from family or community members about what care it is like to give birth in a public facility (Centro de Salud, Hospital Nacional, o CUM)?

- Have you heard any negative comments? What did they say?
- Have you heard any positive comments? What did they say?
- Do you trust these opinions a little or a lot?
- Did these comments influence your feelings about having your baby in a public facility?

1. This pregnancy when you needed to visit the hospital/clinic, did you go with the care navigator from the organization Wuqu’ Kawoq? This is the “nurse” that stayed with you while in the hospital/clinic. If so, you don’t need to tell me the name of the which care navigator was with you.
2. Did she speak to you in Spanish or Kaqchikel?
3. How did she assist in you getting the care you needed?

- Were there things she did that were helpful or prevented harm?
- Were there things she did that were not helpful or harmful?
- For example, did she help get a medication or translate what the doctors/nurses said to you?

1. Did you give birth during this hospital stay? *If yes, continue to sub-questions*

- Was it a normal delivery or surgery (Cesarean)?
- What position were you in when you delivered?
- Was the care navigator present when you delivered?

1. Overall, how was your experience during that referral?

- Why?
- Did you feel more or less comfortable with her to accompany you in the hospital?

1. Were you able to communicate well with the doctor or nurse?

- How so?
- Did you feel you more or less able to communicate with the doctors and nurses with her (the care navigator) there?

1. Did you understand the care or recommendations you received from the doctors?

- If not, did you feel able to ask questions?
- Why or why not?

1. Did you feel any negative feelings about the hospital experience, such as fear or being judged?

- Do you think that the doctor/nurses treated you better or worse with her there?

1. Did you feel any positive feelings about the hospital experience, such as being supported?

- Do you think that the doctor/nurses treated you better or worse with her there?

1. Do you think the experience with the care navigator changed how you think about the hospital care?
2. Would you be willing to go to the hospital during your next pregnancy?

- Why or why not?

1. If you needed to go to the hospital during your next pregnancy, would you want her (the care navigator) to go with you?

- Why or why not?

Thank you for taking the time to talk with me today. I realize the topics we talked about may have been difficult. If you would like to talk to a doctor about feeling of depression, loneliness, or anxiety, you can contact the Estrellas project and they will connect you with resources in the Health Center in Paquip. If the doctor in the Health Center in Paquip decides you need to be referred we can help with transportation costs. You can call or text Yoli from the Estrellas project at 3027-9461.

**Hospital Staff Interview Guide**

Obstetric Care Navigator Pilot Program Qualitative Study

Interview date:

Interviewer:

Language:

Consent to interview obtained?

Recorded or written?

I appreciate your willingness to talk with me today about your experience caring for mothers in public hospitals and clinics. Remember, I will not write down your name with your answers so everything you say will be confidential and will in no way affect your employment.

1. Can you tell me a little about yourself?

- What language do you speak at home?
- Where did you grow up?

1. What is your official role here at this Ministry of Health hospital/clinic?

- How long have you worked here?
- What is your schedule like? How many hours do you work daily and how many days a week?)
- *If doctor or nurse:* Where did you study for your degree in nursing/medicine?
- Before this did you work in health care in another setting?

1. What types of services do you offer here at this hospital/clinic for pregnant women?
2. What types of patients do you care for here in maternity?

- Where do they live and travel from?
- What language or languages do they speak?

1. What do you think determines whether a mother is happy with the care she receives at the hospital? We want you to provide specific examples whenever possible, but not the names of specific people involved in that example.

- How are these the same or different for Maya and ladina women? Why or why not?

1. What challenges do you face in taking care of patients here in this hospital/clinic?

- What material or human resources—if any—are lacking?
- What type of patients—if any—are most difficult to care for?
- What unique challenges—if any—have you faced caring for Maya women?

1. In your experience, what are the reasons that women may not agree to a hospital referral? We want you to provide specific examples whenever possible, but not the names of specific people involved in that example.

- Which of these is most important in your observations?
- Which of these have to do with the individual woman? Which ones have to do with the hospital system?
- Which of these do you think the hospital/clinic could adapt to overcome?

1. Now I’m interested in your views on the care navigators, the women that accompany pregnant patients here and offer accompaniment and translation.

- Have you heard about the care navigators?
- Have you personally interacted with the care navigators?

*If interviewee has personally interacted with the care navigators continue here*

*If interviewee has heard about the care navigators but not personally interacted with them skip to question XX*

*If the interviewee has not heard about nor interacted with the care navigators skip to question XX (no additional questions for this sub-group)*

**Personal Interaction**

1. In what ways have you interacted with the care navigators?

- How many times have you interacted with the care navigators?
- There are 3 care navigators, have you interacted with more than one?
- What types of tasks have you observed them perform?

1. As you understand it, what is the purpose and role of the care navigators?

- Are these tasks important? Why or why not?
- Is this role needed? Why or why not?

1. Do you think the care navigators have an impact on the experience of women presenting for care during pregnancy?

- What is that impact? Is it positive or negative?

1. What specific benefits—if any—have you seen the care navigators provide to patients? We want you to provide specific examples whenever possible, but not the names of specific people involved in that example.

- What other tasks do you wish the care navigators provided, if any?

1. What specific problems—if any—have you seen the care navigators cause? We want you to provide specific examples whenever possible, but not the names of specific people involved in that example.

- What tasks or activities do you wish the care navigators would avoid, if any?

1. Have you witnessed or heard about any unprofessional behavior by a care navigator toward a patient? You do not need to provide the name of which care navigator in any examples you provide.

- Did you witness or hear about this?

1. Have you witnessed or heard about any unprofessional behavior by a care navigator toward a staff member? You do not need to provide the name of which care navigator in any examples you provide.
2. How—if at all—does the presence of care navigators change how hospital/clinic staff treat patients?

**Heard Only**

1. As you understand it, what is the purpose and role of the care navigators?

- Are these tasks important? Why or why not?
- Is this role needed? Why or why not?

1. Do you think the care navigators have an impact on the experience of women presenting for care during pregnancy?

- What is that impact? Is it positive or negative?

1. What specific benefits—if any—have you heard that the care navigators provide to patients?

- What other tasks do you wish the care navigators provided, if any?

1. What specific problems—if any—have you heard that the care navigators cause? We want you to provide specific examples whenever possible, but not the names of specific people involved in that example.

- What tasks or activities do you wish the care navigators would avoid, if any?

1. Have you witnessed or heard about any unprofessional behavior by a care navigator toward a patient? We want you to provide specific examples whenever possible, but not the names of specific people involved in that example.

- Did you witness or hear about this?

1. Have you witnessed or heard about any unprofessional behavior by a care navigator toward a staff member? We want you to provide specific examples whenever possible, but not the names of specific people involved in that example.
2. How—if at all—does the presence of care navigators change how hospital/clinic staff treat patients?

24) Is there anything else you would like to add or share?

Thank you for your time, we very much appreciate your views and opinions and will ensure your responses are kept anonymous.
